# A Simple High-Content Cell Cycle Assay Reveals Frequent Discrepancies between Cell Number and ATP and MTS Proliferation Assays

**DOI:** 10.1371/journal.pone.0063583

**Published:** 2013-05-17

**Authors:** Grace Ka Yan Chan, Tracy L. Kleinheinz, David Peterson, John G. Moffat

**Affiliations:** 1 Department of Biochemical and Cellular Pharmacology, Genentech, Inc. South, San Francisco, California, United States of America; 2 Department of Discovery Oncology, Genentech, Inc. South San Francisco, California, United States of America; Instituto Nacional de Cardiologia, Mexico

## Abstract

In order to efficiently characterize both antiproliferative potency and mechanism of action of small molecules targeting the cell cycle, we developed a high-throughput image-based assay to determine cell number and cell cycle phase distribution. Using this we profiled the effects of experimental and approved anti-cancer agents with a range mechanisms of action on a set of cell lines, comparing direct cell counting *versus* two metabolism-based cell viability/proliferation assay formats, ATP-dependent bioluminescence, MTS (3-(4,5-dimethylthiazol-2-yl)-5-(3-carboxymethoxyphenyl)-2-(4-sulfophenyl)-2H-tetrazolium) reduction, and a whole-well DNA-binding dye fluorescence assay. We show that, depending on compound mechanisms of action, the metabolism-based proxy assays are frequently prone to 1) significant underestimation of compound potency and efficacy, and 2) non-monotonic dose-response curves due to concentration-dependent phenotypic ‘switching’. In particular, potency and efficacy of DNA synthesis-targeting agents such as gemcitabine and etoposide could be profoundly underestimated by ATP and MTS-reduction assays. In the same image-based assay we showed that drug-induced increases in ATP content were associated with increased cell size and proportionate increases in mitochondrial content and respiratory flux concomitant with cell cycle arrest. Therefore, differences in compound mechanism of action and cell line-specific responses can yield significantly misleading results when using ATP or tetrazolium-reduction assays as a proxy for cell number when screening compounds for antiproliferative activity or profiling panels of cell lines for drug sensitivity.

## Introduction

Plate-based proliferation assays are a fundamental tool in oncology drug discovery for evaluating potency of compounds and sensitivity of different cell lines to specific agents. Historically, direct measurements of cell number have not been practical with high-throughput microtiter plate-based assays, especially with high-density 384 and 1536-well plates. Therefore the most common approach to evaluating either cell number, cell proliferation, and cell viability, depending on the investigator’s point of view, is to measure the per-well amount some aspect of cellular metabolism or biomass as a proxy for the number of viable cells. A thorough review of these and other indirect assays for cell number was recently presented by Quent et al [1]. In this study we will focus on three of the most common methods; determination of ATP in cell lysates by luciferin/luciferase-generated bioluminescence (e.g. CellTiter-Glo (Promega), ATPlite (Perkin Elmer)) [2]–[4], reduction of tetrazolium salts such as MTS and MTT to formazan by cellular dehydrogenases, (mitochondrial and otherwise) [3], [5]–[7], and determination of the total amount of nucleic acid per well fluorescent dsDNA-binding cyanine dyes (e.g. CyQuant (Invitrogen), picoGreen (Invitrogen)) [8]. As commonly used, these assays do not determine absolute mass amounts or molar concentrations of the analytes, but yield signals that have been demonstrated to have a wide dynamic range and linear response within relevant analyte concentrations.

Using these assays, both potency (commonly EC_50_ or IC_50_) and efficacy (percent reduction of response relative to untreated controls, E_max_) are critical parameters for interpreting compound dose-response curves, for example differences on E_max_ can be sufficient to distinguish cytostatic and cytotoxic mechanisms of action (MoA).

Central to interpreting these data in terms of treatment effects on “proliferation”, “cell number” or “viability” is the assumption that a linear relationship exists between cell number and signal, i.e. the amount of analyte or activity per cell remains invariant. However, this assumption is not always justified. For example, a compound that increased cell size without altering the cytoplasmic concentration of ATP would appear to be less efficacious in an ATP assay than it’s actual effect on cell number. This possibility is supported by evidence in the literature for complex regulation of cellular energy metabolism and mitochondrial function during apoptosis and in response to treatment with a variety of cancer drugs [9]–[14].

As opposed to these proxy assays, microscopy and high-content assays using DNA-binding stains to visualize cell nuclei enable direct determination of cell number, avoiding these potential confounding factors. Furthermore, visualization and quantitation of nuclear intensity and morphology is a rich source of information regarding compound MoA, especially for treatments that affect the cell cycle and cell survival. Understanding mechanisms of action is critical for optimizing drug candidates, since off-target activities including, but not limited to, cytotoxicity are a frequent confounding factor in assays. Further, in profiling sensitivity of a panel of cell lines to a specific agent of interest, the phenotypic responses of different cell lines to both on-target and off-target activity can be both informative and confounding.

We report here the development and implementation of a simple no-wash image-based assay to simultaneously determine absolute cell number and cell cycle phase distribution of adherent or suspension cells in 384-well plates. Using this assay we can readily differentiate MoAs of different agents on the same cell line, the same agent on different cell lines, and critically, demonstrate that it is not uncommon for a single drug to have different MoAs at different concentrations.

Using the direct cell count data we demonstrate that drug MoA and cell line variation can both contribute to significant underestimation of potency and/or maximal efficacy when using ATP or MTS-reduction assays as compared to the actual number of cells present in the well. While similar observations have been made before with specific compounds comparing different indirect assay formats, we systematically surveyed a panel of cell-cycle-targeting and chemotherapeutic agents representing multiple mechanisms of action. We also sought mechanistic explanation for these observations. This analysis shows that the inter-assay-format discrepancies are associated with changes in cytoplasmic volume and mitochondrial mass induced by drugs with different cell cycle-targeting MoAs. Therefore, understanding of the drug’s MoA, or at least awareness of the potential impacts of different MoAs on assay readout, is crucial to choosing an appropriate assay strategy and ensuring accurate data analysis and interpretation.

## Materials and Methods

### Cell Culture

Cell lines were obtained from ATCC (Manassas, VA) and maintained in complete growth medium: RPMI supplemented with 10% fetal bovine serum (Sigma; St. Louis, MO) and 1X GlutaMAX™ (Life Technologies; Carlsbad, CA).

### Compound Treatment

Inhibitors were obtained in-house and from commercial vendors: aphidicolin, cisplatin, doxorubicin, etoposide, nocodazole and vinblastine (EMD Chemicals; Philadelphia, PA), gemcitabine (Tocris, Ellisvile, MO), paclitaxel (Life Technologies), 5-fluorouracil (Sigma). All other compounds were synthesized at Genentech. Cells were seeded in 384-well plates at the appropriate density for each cell line in 45 µl medium and left at room temperature (RT) for 30 minutes before incubating at 37**°**C overnight to attach. The pre-incubation at RT minimizes thermal gradients while the cells are settling to the bottom of assay plates, thus allowing a more even distribution of cells within the well [15]. Compounds were serially diluted in DMSO and further diluted to 16x final concentration in medium before 3 ul compound was added to the cells. Final DMSO concentration was 0.25%. Cells were incubated with compounds at 37**°**C for 1–3 days with no further changes of media or re-addition of compounds.

### ‘Proliferation’ Assays

*CellTiter-Glo assay (Promega):* Measurements were made according to manufacturer’s instructions. Briefly, plates were removed from the incubator and allowed to equilibrate at room temperature for 20 minutes, and equal volume of CellTiter-Glo reagent was added directly to the wells. Plates were incubated at room temperature for 30 minutes on a shaker and luminescence was measured on an Envision reader (PerkinElmer). Luminescence reading was normalized to and expressed as a relative percentage of the plate-averaged DMSO control.

*CellTiter-AQueous MTS assay (Promega):* Measurements were made according to manufacturer’s instructions. Briefly, 10 µl of MTS reagent was added directly to the wells and cell plates were incubated at 37**°**C for a minimum of 1 hour. Absorbance was measured at 490 nm on a SpectraMax Plus384 reader (Molecular Devices; Sunnyvale, CA). Background absorbance was first subtracted using a set of wells containing medium only, then normalized to and expressed as a relative percentage of the plate-averaged DMSO control.

*CyQUANT direct assay:* Measurements were made according to manufacturer’s instructions (Life Technologies). 2X detection reagent was prepared by adding the supplied direct nuclei acid stain and direct background suppressor I in cell culture media. Equal volume of this 2X detection reagent was then added directly to the wells and cell plates were incubated at 37**°**C for 1 hour. Fluorescence was measured at 508 nm excitation and 527 nm emission on a Infinite® M1000 PRO reader (Tecan; Männedorf, Switzerland). Background fluorescence was first subtracted using a set of wells containing medium only, then normalized to and expressed as a relative percentage of the plate-averaged DMSO control.

### FACS Cell Cycle Analysis

HT29 cells were seeded in 10 cm dishes and left to attach overnight at 37**°**C. Medium was aspirated off and replaced with medium containing the appropriate concentration of compound. Cells were further incubated with compounds for 24 hours at 37**°**C before being harvested, washed twice and resuspended in 2 ml of PBS containing 0.1% bovine serum albumin (BSA). Cells were fixed with cold 70% ethanol for at least 1 hour at 4**°**C. After 2 washes with PBS, cells were resuspended in 2 ml of propidium iodide (PI)/RNase staining solution (Cell Signaling Technology; Danvers, MA) and incubated for at least 3 hours at 4**°**C. Cells were analyzed with BD FACSCalibur™ flow cytometer (BD Bioscience; Franklin Lakes, NJ). The PI fluorescence signal at FL2-A peak versus counts was used to determine cell cycle distribution.

### High-content Cell Cycle Assay

After compound treatment, cells were fixed, permeabilized and nuclei-stained in one step by adding equal volume of phosphate buffered saline containing 2x final concentration of 0.25% paraformaldehyde (Electron Microscopy Sciences; Hatfield, PA), 0.075% saponin (Sigma) and 2 µg/ml Hoechst 33342 (Life Technologies) directly to the wells. Cell plates were incubated in the dark at room temperature on a shaker for 30 minutes before imaging on Opera**®** high content screening system (PerkinElmer Inc). Images were acquired using a 10x water immersion objective and the non-confocal UV channel. Six image fields were recorded for each well, corresponding to approximately 40% of the area of the well.

### Mitochondria Detection

Live cells were incubated with 100 nM MitoTracker Deep Red FM (Life Technologies) with or without 200 nM TMRE (tetramethylrhodamine ethyl ester, Life Technologies) at 37**°**C for 30 minutes. Cells can then be imaged live at this point or in our case, fixed and stained as described above to allow simultaneous cell cycle analysis. Confocal mages were acquired using the Opera instrument simultaneously with the DNA fluorescence images as described above using a 635 nm excitation laser and 690/50 emission dichroic for MitoTracker Deep Red and 532 excitation and 585/50 emission for TMRE.

### Measurement of Oxygen Consumption Rates

Cells were plated at 20,000 cells per well in XF 96-well cell culture microplates (Seahorse Bioscience) pretreated with poly D lysine and incubated for 24 h at 37**°**C in a 5% CO2 incubator. To assay oxygen consumption rate (OCR) and extra cellular acidification rate, the growth media was replaced with bicarbonate-free, serum-free pre-warmed medium and the plate was loaded into the XF96 Extracellular Flux Analyzer (Seahorse Bioscience). OCR baseline measurements were determined for the HT29 cell lines pretreated with anti-cancer agents for 24 hrs. Measurements of baseline OCR and ECAR were taken and cells were treated in succession with 1 uM Oligomycin (Cell Signaling) and then 1 uM FCCP (Abcam). Cell numbers used for normalization were determined by fixing the plate after analysis with 4% paraformaldehyde, staining with Hoechst, imaging 4 quadrants/well on a Molecular Devices ImageXpress HCS, and counting the average nuclei number per quadrant.

### Data Analysis

Images were analyzed with a custom script written in Acapella® (PerkinElmer Inc.). Image segmentation to identify nuclei and calculation of integrated pixel intensity for each object in the Hoechst emission channel were performed with the optimal “detect nuclei” module. Cell (nuclei) number was then normalized to, and expressed as, a percentage of the plate-averaged DMSO control. For cell cycle distribution analysis, integrated Hoechst fluorescence intensity was first log_2_ transformed. For each experiment, log_2_ intensity histograms from multiple DMSO control wells were first analyzed to determine the intensity value corresponding to the center of the 2N sub-population. This value was then applied as an input parameter to define a search range for the exact 2N DNA peak for each well and to normalize DNA intensity to this value, such that the maximum of the 2N peak corresponded to 1 and the center of the 4N DNA peak corresponded to 2. Individual cells were then categorized according to DNA content; sub-G1(log2 normalized DNA intensity<0.75), 2N (0.75–1.25), S (1.25–1.75), 4N (1.75–2.5) and >4N (>2.5). The percentage of cells in each phase per well was then output. For mitochondrial features, the MitoTracker stain was used to define a cytoplasmic region around each detected nuclei using the Acapella “detect cytoplasm” module, and the area, mean intensity and integrated intensity determined for each cell. The mean value of the integrated MitoTracker and TMRE intensities for all the imaged field in the well was calculated then normalized to and expressed as a fold change relative to the plate-averaged DMSO control.

Dose-response curves for cell count, ATP and MTS assays were analyzed using the Condeseo module of Genedata Screener (GeneData AG). The Robust fit (Tukey biweight) strategy was used to fit data to a 4-parameter logistic fit with the following restrictions: 20<span<100, 0.5<Hill Slope<4. Acceptance criteria for valid fits were √χ^2^/F <(1.5×global std deviation) and S.E. Log EC_50_<1.

## Results

### Cell Cycle and Cell Number Assay Development and Validation

To study potency and mechanisms of action of compounds with predicted antiproliferative and cell cycle-mediated effects, we optimized a procedure for staining and imaging fixed cells in a high-throughput manner. Quantitation of dead/detached as well as viable cells is desirable when profiling potentially apoptosis-inducing or cytotoxic agents. To that end we developed a protocol without any washes or changes of medium. Cell cycle phase classification was achieved by DNA histogramming, therefore maintaining a linear relationship between DNA content and integrated DNA intensity was critical. This was achieved using mild detergent permeabilization of fixed cells to facilitate uniform Hoechst staining.

**Figure 1:**
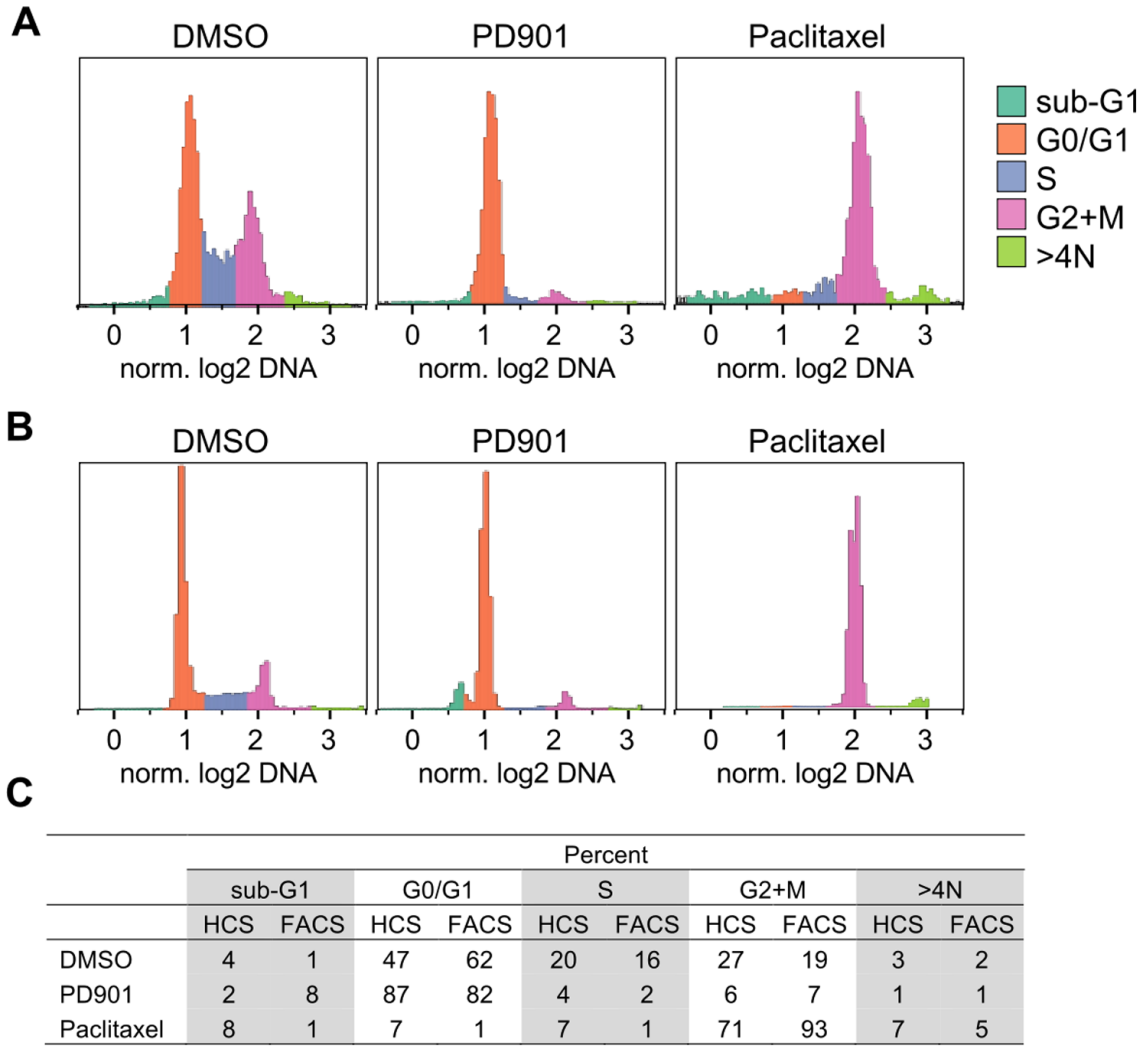
High-content cell cycle analysis. HT29 cells treated for 48 hours with the indicated treatments in a 384-well plate were fixed and stained with Hoechst 33452, imaged, and integrated staining intensity of individual nuclei was estimated as described in Materials and Methods. **A**. DNA content histograms for log-2 transformed DNA intensity values normalized to the modal value of the 2N DNA. Automatic classification into sub-G1, 2N, S-phase, 4N and 8N populations is indicated. **B**. DNA content histograms acquired by flow cytometry, raw FL2-A data was normalized and binned in the same way as high-content data. **C**. Quantitation of the sub-population fractions of the histograms.

To verify that quantitation of DNA content was linear, HT29 cells were treated with drugs inducing specific cell cycle arrest phenotypes as shown in figure 1. A MEK kinase inhibitor, PD901 induced cell cycle arrest at the G1/S checkpoint (2N DNA content) through upregulation of p27 and downregulation of cyclin D1 [16], [17], the antimitotic drug paclitaxel caused mitotic arrest (4N DNA), while the Aurora kinase inhibitor VX-680, which is known to cause endoreduplication [18], yielded a population of cells with 8N DNA content. Figure 1A shows that for histograms of log2-transformed integrated DNA intensity, the expected two-fold (one unit) increases in peak intensity between the centers of 2N, 4N and 8N peaks were observed. The “gold standard” for determination of DNA content is flow cytometry; comparison data illustrated in figure 1B shows that the main difference is a broadening of 2N and 4N peaks with the image-derived intensities, and correspondingly the absence of a distinct intermediate S-phase population, however if the same binning rules are applied to both sets of data the sub-population frequencies under control and drug-treated conditions are comparable.

### Determination of Compound Mechanisms of Action from Cell Cycle Analysis

Dose-dependent changes in the number of cells and in the cell cycle population distributions were measured simultaneously by the method described above. Initial assay validation used HT29 cells treated for 48 hours. This cell line was selected because the presence of the B-Raf(V600E) mutation confers the ability to induce a cytostatic G1 arrest with MAPK pathway kinase inhibitors, and because their morphology is suitable for image analysis (i.e. growth as a monolayer with individual nuclei and cytoplasm robustly detected by automated image segmentation).

**Figure 2:**
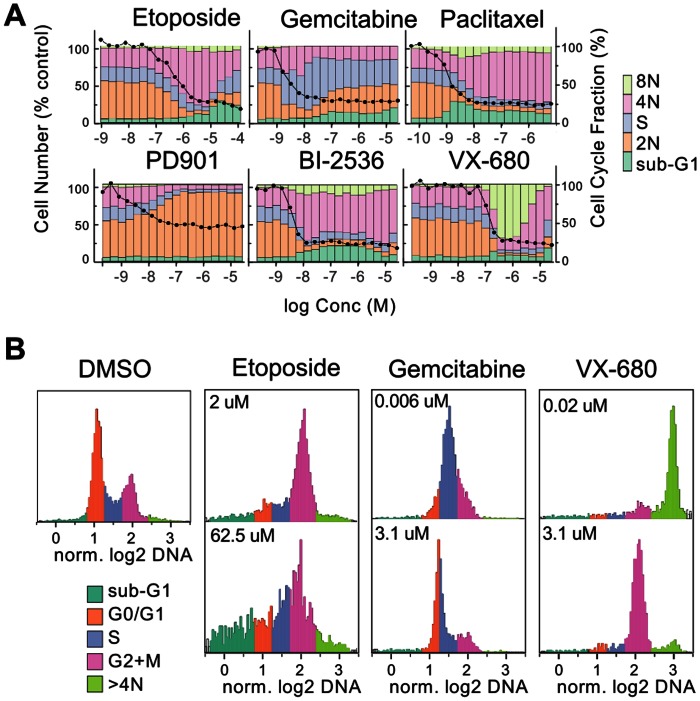
Cell cycle profiles derived from high-content assay. Cells in the same images used for direct cell counting were classified into five cell cycle bins by integrated DNA intensity. **A.** Stacked bars show the relative frequencies of the sub-populations at the indicated concentrations. Each bar is the average of two wells. Black circles indicate relative cell number. **B.** Histograms of integrated DNA intensity derived from the images from individual wells showing the change in cell cycle profile from EC_90_ (upper panels) to higher doses of etoposide, gemcitabine and VX-680.

A set of chemotherapeutic agents and kinase inhibitors with known or predicted activity against specific points in the cell cycle were tested, as summarized in table 1. Figure 2A shows the dose-dependent changes in cell number and population fractions for a subset of these compounds. The cell cycle sub-population profiles for all the other compounds tested are in Figure S1.

**Table 1:** Comparison of assay results for HT29 cells.

		Log EC_50_ (M)			E_max_ (% Reduction)			
Treatment	Target	Count	ATP	MTS	Count	ATP	MTS	Cell Cycle Response
Cytochalasin D	Actin cytoskeleton	−7.3	−7.1	−7.1	79	**44**	**47**	Sub-G1
Aphidicolin	DNA Polymerase	−6.9	***>−4.6***	***>−4.6***	77	***<20***	***<20***	4N, S
Doxorubicin	Topoisomerase II	−7.7	***−*** **6.5**	***−*** **6.6**	85	**58**	**45**	ND
Etoposide	Topoisomerase II	−6.8	***−*** **4.5**	***−*** **4.6**	79	80	74	4N, sub-G1
Gemcitabine	DNA Synthesis	−8.7	***>−4.6***	−**7**	74	***<20***	**26**	S
5-FluoroUracil	Nucleoside Synthesis	−5.7	***−*** **3.8**	−5.7	79	**55**	61	S
Cisplatin	DNA Alkylation	−5.4	***−*** **4.1**	***−*** **4.2**	77	>60	>70	4N
Trichostatin A	HDAC	−7.4	−7.3	−7.3	93	97	79	Sub-G1
BI-2536	PLK1	−8.7	*ND*	*ND*	86	*ND*	*ND*	4N
SNS-032	Cdk2/7/9	−7.3	−7.4	−6.9	85	**51**	**43**	4N
ARQ-197	Met	−6.2	−6.2	−6.1	88	**37**	**39**	4N
PD901	MEK	−8.7	−8.2	−8.1	62	53	50	2N
Crizotinib	Met/ALK	−6.1	−5.8	−5.5	88	98	82	4N, sub-G1
Purvalanol A	Pan-Cdk	−5.4	−5.1	−4.9	89	**57**	73	4N
Staurosporine	Pan-kinase	−8	−7.3	−7.7	90	97	**45**	4N, sub-G1
VX680	Aurora B	−7.3	*ND*	*ND*	77	*ND*	*ND*	8N, 4N/sub-G1
Actinomycin D	RNA Polymerase	−8.8	−8.2	−9.7	89	**62**	**48**	4N/sub-G1
Brefeldin A	Golgi Traffic	−7.5	−7.4	−7.3	87	91	65	Sub-G1
Colcemid	Microtubule destab	−7.9	−8	−7.8	84	**51**	**46**	4N
Colchicine	Microtubule destab	−8.3	−7.9	−7.8	84	**49**	**47**	4N
Epothilone B	Microtubule stab	−8.6	−8.3	−8.2	78	**51**	**41**	8N/4N
Nocodazole	Microtubule destab	−8	−7.9	−**7**	87	**41**	**42**	4N
Paclitaxel	Microtubule stab	−8.9	−8.8	−9.1	87	66	**37**	4N
Vinblastine	Microtubule destab	−8.7	−8.5	−8.4	83	**52**	**49**	4N
Vincristine	Microtubule destab	−8.8	−8.6	−8.4	84	**54**	**45**	4N

Values in bold indicate ATP or MTS assay log EC_50_ differing by >1 log unit from cell count EC_50_, or ATP or MTS assay E_max_ differing by >25% from the cell count E_max_. ND, valid curve fits could not be obtained according to the criteria described in Materials and Methods.

Most compounds showed cell cycle profile changes, in keeping with their expected MoAs, coinciding with the decrease in cell number. For example, paclitaxel induced a robust mitotic arrest (4N DNA content) across a broad concentration range. Similar results were found for another microtubule-stabilizing drug, epothilone B, and for the microtubule-destabilizing agents nocodazole, colcemid and vinblastine (Figure S1).

However a number of compounds, exemplified in figure 2 by etoposide, gemcitabine, VX-680 and BI-2536, showed further changes in cell cycle profile at higher concentrations, giving biphasic dose-responses. DNA content histograms in figure 2B illustrate in more detail the switching from the profile at ∼EC_90_ to the response at higher concentrations. At cell count EC_90_ etoposide shows a predominant G2 arrest, while at 62 µM there is significantly more heterogeneity and a larger sub-G1 fraction. In the range of 10–100 nM gemcitabine, an S-phase population predominates but at higher concentrations the histogram shows a shift to arrest earlier in S-phase While VX-680 is an inhibitor of Aurora A and B, the phenotypic response is typically consistent with Aurora B inhibition [18]. The accumulation of cells with 8N (and higher ploidy) DNA content shown in figures 1 and 2 is typical. The second-step decrease in ATP and MTS coincided with a change in the cell cycle profile from predominantly 8N to larger 4N and sub-G1 fractions (Figure 2B). In other cases where there was a change in the dominant phenotype at different concentrations, e.g. cisplatin, staurosporine and the cMet/ALK kinase inhibitor crizotinib [19] all showed a transition from 4N DNA (G2 or M) to a significant sub-G1 (nuclear fragmentation/apoptosis) population at higher test concentrations.

The PLK1 inhibitor BI-2536 has been reported to cause prometaphase arrest, followed by mitotic catastrophe and apoptosis, in HeLa and HCT116 cells [20]–[22]. In the case of HT29 there was a predominant 4N (prometaphase) population but very little sub-G1 population at the 48 hour timepoint.

The value of this method in detecting unexpected off-target effects of compounds was demonstrated by the observation that the putative cMet kinase inhibitor ARQ-197 (tivantinib) [23] inhibited cell proliferation and induced a M-phase arrest (Figure S1), which is not the phenotype expected for cMet inhibition. This observation is consistent with a recent report that tivantinib inhibits tubulin polymerization [24].

### Comparison of Assay Formats

The high-content assay thus enabled a direct comparison of compound potency and efficacy as determined by direct cell counting *versus* the ATP-dependent luciferase/luciferin and MTS-reduction assays. A total DNA fluorescence assay (CyQuant) was also compared.

To compare the different assay formats, replicate plates were treated with serial dilutions of each compound for 48 hours. 20-point two-fold serial dilutions were carried out to ensure that a complete range of responses would be observed. One replicate plate was then processed for each of the standard ATP CellTiter-Glo assay, MTS colorimetric assay, CyQuant and the high-content assay as described above. Dose-response curves for cell number and luciferase, MTS and CyQuant assay signals were analyzed by fitting to a 4-parameter logistic model with unconstrained upper and lower asymptotes and fit acceptance criteria as defined in the methods section. EC_50_ (concentration giving 50% of maximum response) and E_max_ (maximum percent signal reduction as defined by lower asymptote of fit curve) values from these curve fits are summarized in table 1.

**Figure 3:**
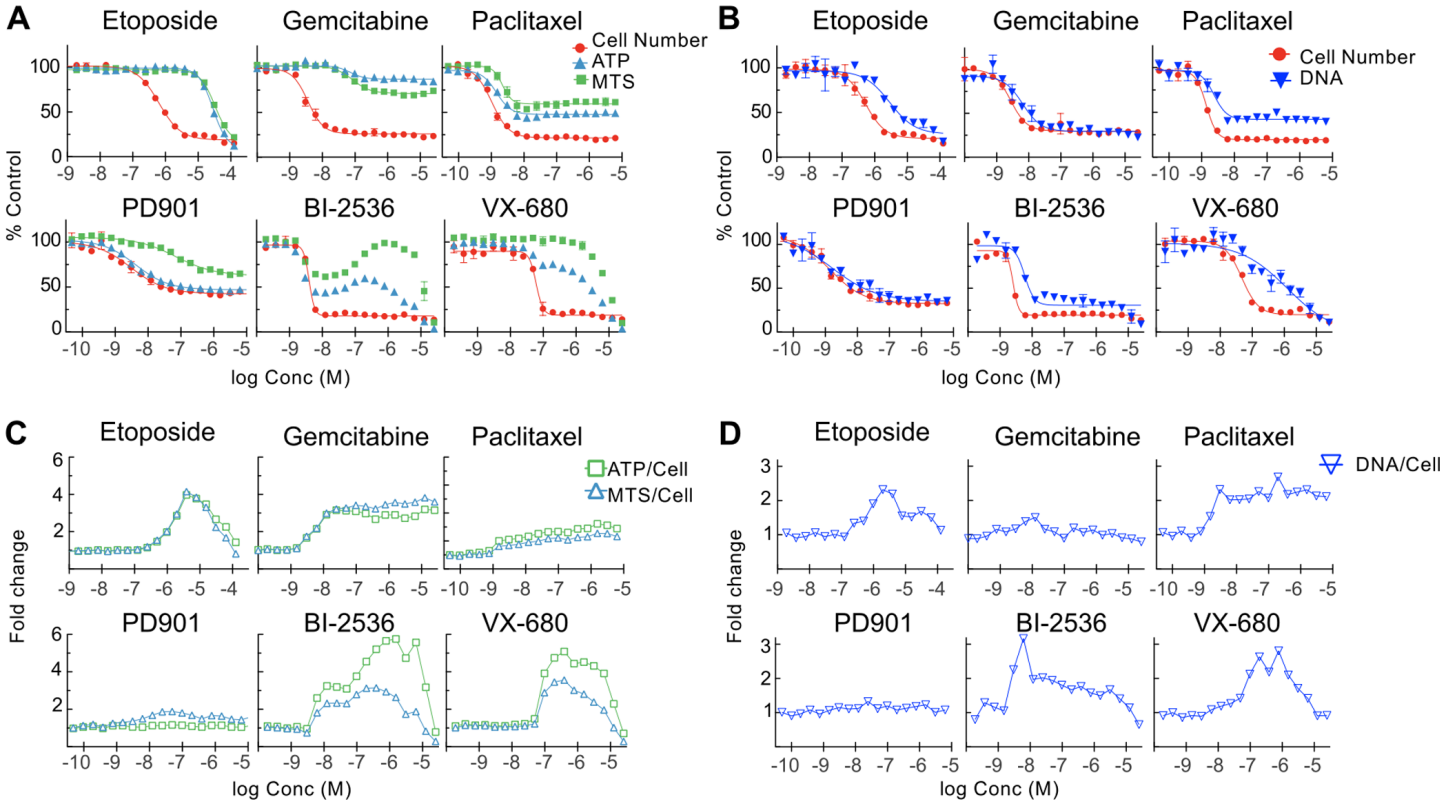
Comparison of ATP and MTS assays with direct cell counting. Replicate plates of HT29 cells were treated as indicated for 48 hours then analyzed by ATP or MTS assay or high-content cell counting. **A.** Normalized values for direct cell number (red circles), ATP assay (RLU) (blue triangles) and MTS assay (E_490_) (green squares), lines indicate fits to 4-parameter logistic model. If no line is shown then regression did not result in a curve that met acceptance criteria. **B.** Normalized values for direct cell number (red circles), and DNA assay (CyQuant) (blue triangles) **C.** Fold change in normalized ratios of ATP RLU signal to cell number (green squares) and MTS assay signal to cell number (blue triangles)**D.** Fold change in normalized ratio of DNA assay signal to cell number.

As table 1 shows, the degree of agreement between the cell number and metabolism-based proxy assay results varied significantly between compounds. While EC_50_ values derived from cell number were in most cases comparable to the ATP and MTS assay results, the DNA synthesis-targeting agents were striking exceptions. Further, some treatments (e.g. the mitotic kinase inhibitors BI-2536 and VX-680) yielded non-monotonic dose response curves with the ATP and MTS assays that could not be fitted to valid curves (see figure 3A), although the cell number dose-response curves were well-behaved.

The efficacy, or E_max_, results showed greater differences than EC_50_ values between assay formats for many of the treatments. Typically, although the cell numbers were reduced by about 80% at maximally efficacious concentrations, there was significantly less reduction in ATP and MTS signal; for example all the microtubule-targeting agents gave E_max_ values in the range of 45–60% reduction. In the extreme cases of aphidicolin and gemcitabine, the reduction in ATP and MTS signal was insufficient for valid curve fits, despite ∼80% reductions in cell number.

The dose-response curves for selected compounds that showed significant differences between assays are presented in figures 3A and 3B. The deviations between cell number and ATP or MTS assay signals (figure 3C) and total DNA (figure 3D) are illustrated as fold change in the normalized ratio of signal to cell number. Curves for the other compounds listed in table 1 are presented in Figure S2. Gemcitabine, a nucleoside analog, and etoposide, a topoisomerase II inhibitor, along with aphidicolin and cisplatin, which also inhibit DNA replication, represent the class of compounds that showed the most striking discrepancies between the assays. The ATP and MTS curves for etoposide were right-shifted by greater than 10-fold relative to the cell number, but converged to a similar E_max_ value at maximal concentration. Thus the ratios of ATP and MTS per cell showed a bell-shaped response with a maximal increase of 4-5-fold. Gemcitabine induced an increase in ATP and MTS per cell of similar magnitude, but in this case the elevation was constant, and the ATP and MTS curves did not decrease or converge with the cell number, up to the highest concentration tested (25 µM).

The discrepancies between the different dose-response curves for paclitaxel (figure 3) and other microtubule-targeting drugs (Figure S2) were not as dramatic as for the DNA-targeting agents. While the EC_50_s for MTS and ATP curves were not shifted relative to cell number, the E_max_ values were significantly less (45% and 55% respectively) than the 85% reduction in absolute cell number. This corresponded to a 2-fold increase in ATP/cell and MTS/cell.

PD901, which causes G1 arrest phenotype, yielded cell number and ATP curves that were completely superimposable, however the MTS curve was significantly more shallow, corresponding to an approximately 2-fold increase in MTS/cell ratio. The reduction in absolute cell number was less for this treatment than others that were also cytostatic. This is consistent with the fact that PD901inhibits cell division at the G1/S transition, thus any cells in S, G2 or M-phase at the time of exposure to the drug will complete one doubling before arrest.

The effects of two mitotic kinase inhibitors, VX-680 and BI-2536, are also shown in figure 2. The cell number curves showed a monotonic decrease allowing robust EC_50_ determination, but the ATP dose-response curves were considerably more complex. VX-680 gave a 2-step biphasic decrease, with an initial decrease at a similar concentration to the cell number response, followed by a plateau at ∼30% effect before a second decrease. The MTS signal, on the other hand, did not decrease until the same concentration as the second step of the ATP curve. The PLK1 inhibitor BI-2536 also gave ATP and MTS dose-response curves that were significantly different from the cell number, and were striking in their complexity. Both assays showed multiphasic dose-response curves (Figure 3) where the initial signal decrease corresponded to the cell number response, followed by increases before dropping again at higher concentrations.

Data generated using the total DNA fluorescence signal (CyQuant) was also compared with direct cell counting. Unlike the other two proxy assays, this assay signal should be unaffected by changes in cell size or metabolic activity. The presence of a cell-impermeable quenching reagent serves to limit the assay to detecting only cells with intact plasma membranes. Figure 3B illustrates that for the same set of compounds, there was significantly less discrepancy from cell number than the metabolism-based proxy assays. However some treatments, for example etoposide, paclitaxel and VX680, still caused significant differences in E_max_ values between cell number and CyQuant signal. These differences are fully consistent with the changes in average DNA/cell ratio expected for accumulation of cells with 4N or 8N DNA content, plotted as a normalized ratio in figure 3D.

### Similar Effects are Seen Across Multiple Cell Lines

We also wished to determine whether these changes were generalizable to more cell lines. A set of compounds that showed significant inter-assay-format deviations were analyzed in parallel in the high-content, ATP and MTS assays as described above using 5 further cell lines; A375 (BRafV600E, p53wt), A549 (k-ras G12Sp53 wt), HCT116 (k-Ras G12D, p53 wt, PI3Ka H1047R), DLD1 (K-Ras G13D, p53-mut) and NCI-H1299 (N-Ras Q61K, p53-null).

**Figure 4:**
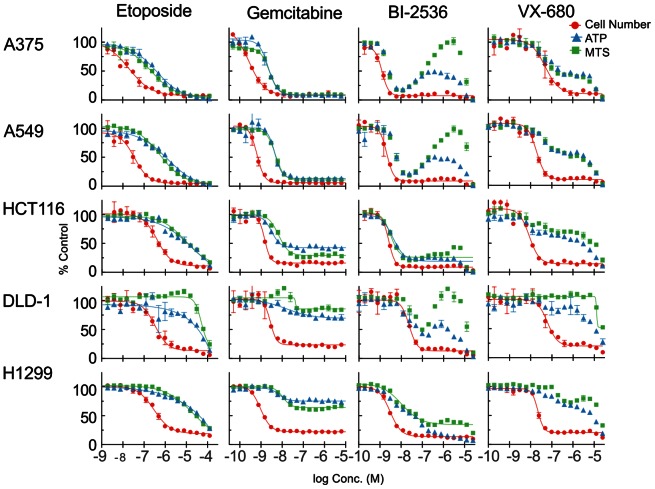
Comparison of ATP and MTS assays with direct cell counting for multiple cell lines. Replicate plates of cells were treated as indicated for 48 hours then analyzed by ATP or MTS assay or high-content cell counting. Normalized values for direct cell number (red circles), ATP assay (RLU) (blue triangles) and MTS assays (E_490_) (green squares), lines indicate fits to 4-parameter logistic model. If no line is shown then regression did not result in a curve meeting acceptance criteria.

Dose-response curves for cell count, ATP and MTS assays for gemcitabine, etoposide, VX-680 and BI-2536 are shown in figure 4. Curve fit results (EC_50_ and E_max_) for these and other compounds are summarized in table S1). The results for etoposide are similar to HT29 for all lines; the most significant difference between the ATP and MTS assays and direct cell count is an underestimation of potency (i.e. right-shifted curves). DLD-1 differs in having a greater shift and a more significant elevation of MTS signal than ATP. However in all cases the ATP and MTS signals reach a similar E_max_ as the cell count. Gemcitabine caused different effects on the ATP/cell and MTS/cell ratios in different cell lines. A549, A375, and HCT116, which are p53-wild type, showed 5-10-fold shifts in EC_50_, with curve E_max_ close to the cell count E_max_ – this corresponds to a transient elevation of ATP/cell and MTS/cell. DLD1 and H1299, which like HT29 are p53-null, exhibited elevated per-cell ATP and MTS, over the entire efficacious concentration range (7nM –25 µM) and thus significantly smaller E_max_. The other DNA synthesis inhibitor tested, Aphidicolin, showed a similar difference in ATP and MTS E_max_ between p53-wt and p53-null cell lines (Table S1).

The cellular responses to VX-680 are consistent with the HT29 data discussed above. In all cases the ATP and MTS curves show a small initial decrease at the same concentrations as the cell number, due to an increase in per-cell ATP and MTS signal. Cell cycle profiles show the same biphasic accumulation of 8N and then 4N fractions at increasing concentrations, as described above for HT29 (data not shown). The PLK1 inhibitor BI-2536 induced the same type of aberrant ATP and MTS curves described above for HT29 with all cell lines except HCT116 and H1299. As summarized in table S1, valid fits could not be obtained for 4/6 cell lines with the MTS assay.

### Simultaneous Determination of Mitochondrial Mass, Cell Number, and Cell Cycle Distribution

The observed increases in ATP per cell imply either an increase in cell size (cytoplasmic volume) with a constant concentration of ATP or a drug-induced increase in ATP concentration and metabolic activity. In order to measure both metabolic (ATP-generating) capacity and cell size, we extended our high-content assay protocol to include staining with MitoTracker Deep Red dye, which accumulates in active mitochondria and is retained upon fixation and mild detergent permeabilization [25], [26]. Thus we were able to quantify mitochondrial mass along with DNA content on a per-cell basis.

**Figure 5:**
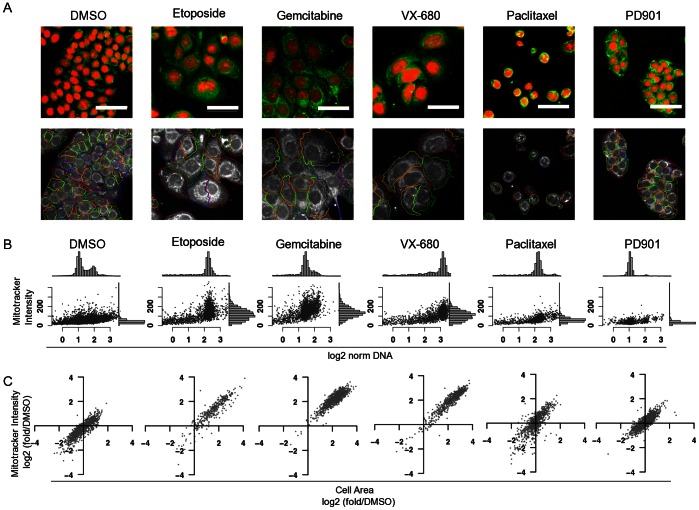
Effects of compound treatment on mitochondrial mass and cell size. HT29 cells were treated with the indicated drugs for 48 hours. (Aphidicolin; 3.1 µM, Etoposide; 3.9 µM, Gemcitabine; 24 nM, Paclitaxel; 98 nM, VX-680; 195, nM, PD901; 390 nM). **A.** Images of Hoechst 33452-stained DNA (red) and MitoTracker deep red-FM staining (green) were acquired with a 20x water-immersion objective as described in Methods section. All images are shown at same magnification and intensity scaling. Scale bar = 50 µm. **B.** MitoTracker deep red images showing identification and segmentation of cell boundaries. **C.** Scatter plots and histograms of log2-normalized integrated DNA intensity (x axis, upper histogram panels) and integrated cytoplasmic MitoTracker Deep Red FM staining intensity (y axis, right-side histograms) derived from the same wells as shown in part A. **D.** Variation in cell area and mitochondrial content (integrated MitoTracker deep red intensity) for the same cell populations, normalized as fold change relative to the mean area and intensity of DMSO-treated samples.

Effects of selected compounds are illustrated in figure 5. A representative image of MitoTracker-stained cells from one of the same wells used to generate the data is shown in figure 5A. Figure 5B shows quantitation of mitochondrial mass plotted as a function of DNA content. Each image and plot was generated from wells treated with concentrations closest to the cell number EC_90_. In some cases striking drug-induced increases in per-cell MitoTracker staining and morphology are evident. Increased mitochondrial mass could be associated with either greater density (e.g. mitochondrial proliferation) or with increases in cell size while maintaining constant density. Figure 5C shows that the latter case was evident with a clear correlation between cell area, determined using the background MitoTracker staining and integrated intensity of MitoTracker. Data for the other compounds tested are presented in figure S3.

Etoposide and gemcitabine treatment resulted in cells with large uncondensed nuclei, the DNA content profiles consistent with arrest at the G2 DNA damage checkpoint for the former and during S-phase for the latter. The Aurora B inhibitor VX-680 induced large multilobed nuclei, predominantly with 8N DNA content. These mechanisms of cell cycle arrest were also associated with greatly increased cytoplasmic and total cell areas, which corresponded to increased mitochondrial content. Plots of cell area versus mitochondrial content for the other test compounds, presented in figure S2, show that other compounds that increased cell size; aphidicolin, BI-2536, doxorubicin, also caused a proportionate increase in mitochondrial content. While paclitaxel and other microtubule-targeting agents also induced robust mitotic arrest, there was neither an observable increase in mean cell area nor mitochondrial content. Cells arrested in G1 by PD901 had no significant change in MitoTracker staining intensity compared to the DMSO controls.

It has been demonstrated previously that mitochondria proliferate continuously and asynchronously throughout the cell cycle to maintain a constant mitochondrial mass per cell at each cell division [27], therefore cells in G2 and M-phase are expected to have a greater mitochondrial content than G1 cells. The data in figure 5B allowed us to assess whether the increases in per-cell ATP and MTS activity were simply due to an increase in the fraction of larger G2/M cells. However, while 4N cells in the untreated samples showed some increase in integrated MitoTracker intensity compared to the 2N population, they still had significantly lower integrated intensity than etoposide-induced 4N and gemcitabine-induced S-phase arrested cells. Therefore accumulation of mitochondrial mass and ATP is a specific response to drug treatment.

### Drug-induced Increases in Mitochondrial Mass Correlate with Changes in ATP:cell ratio

**Figure 6:**
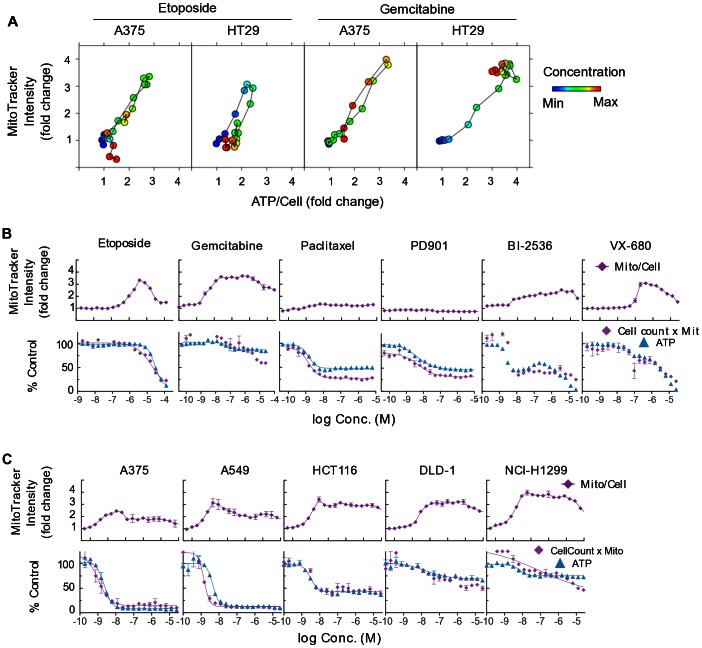
Drug-induced increases in mitochondrial mass correlate with increases in per-cell ATP and MTS assay signal. **A.** Correlation between ATP/cell (RLU/cell number) vs mean per-cell integrated MitoTracker intensity for each point of the dose-response curves. Each point is the mean of replicate wells. Colors indicate relative concentrations in 2-fold dilutions from highest (red) to lowest (blue). Points are connected in order of concentration. **B, C** Upper panels show fold change in mean per-cell integrated MitoTracker staining intensity as a function of concentration. Lower panels show dose-response curves for the total mitochondrial mass, (cell number multiplied by mean per-cell MitoTracker intensity) (purple) overlaid with the ATP assay data (blue). **B.** HT29 cells treated with the indicated compounds. **C.** Different cell lines as indicated treated with gemcitabine.

We next sought to determine whether a quantitative relationship existed between mitochondrial mass increase and changes in ATP/cell and MTS/cell ratio. A direct comparison of the average per-cell integrated MitoTracker intensity versus the per-cell ATP assay signal for etoposide and gemcitabine-treated HT29 cells is plotted in figure 6A. This plot shows that the dose-dependent increases in both per-cell values are highly correlated. In the case of gemcitabine, as observed from the dose-response curve plots, both values increase to a plateau. Etoposide, however, shows correlated increases and subsequent decreases in both values at increasing concentrations, reflecting the biphasic or bell-shaped dose-response curves and the biphasic mechanism of action of this drug.

In figure 6B, the per-cell MitoTracker intensity for HT29 cells treated with the indicated compounds is plotted in the upper panels. The MitoTracker intensity data was generated from the same samples as the cell count and cell cycle data in figures 3 and 5. To establish whether the increase in per-cell mitochondrial mass was sufficient to explain the changes in the ratio of total ATP to cell number, the total per-well mitochondrial mass was calculated by multiplying cell number by average per-cell MitoTracker intensity. Plotting this value yielded dose-response curves for the total per-well mitochondrial mass that closely match the per-well ATP signal (lower panels, figure 6B).

The same analysis is plotted in Figure 6C for the five other cell lines treated with gemcitabine. The cell line-dependent variations in ATP versus cell number responses are mirrored by the changes in total per-well mitochondrial mass.

The EC_50_ and E_max_ results for the total per-well mitochondrial mass dose-response curves shown here, and for a total of eight compounds tested against all six cell lines, are included in table S1, alongside the cell count, ATP and MTS assay data. The total mitochondrial mass results are in good agreement with the ATP assay results.

### Drug-induced Changes in Mitochondrial Activity

Changes in the ATP content of cells could be influenced not only by cell size but also by changes in respiratory activity. Therefore we tested whether the increase in per-cell ATP was correlated with increases in metabolic activity for treatments which did or did not induce corresponding changes in mitochondrial content. HT29 cells treated for 24 hours with selected compounds were analyzed for oxygen consumption rate (OCR) and extracellular acidification rate (ECAR), a measure of glycolytic activity. These values were then normalized to cell number (Figure S4). Replicate plates were analyzed for ATP content, cell number, cell size and mitochondrial mass as described above, however the mitochondria were also stained with the membrane-potential-sensitive dye TMRE to test whether drug treatments were affecting ΔΨ.

**Figure 7:**
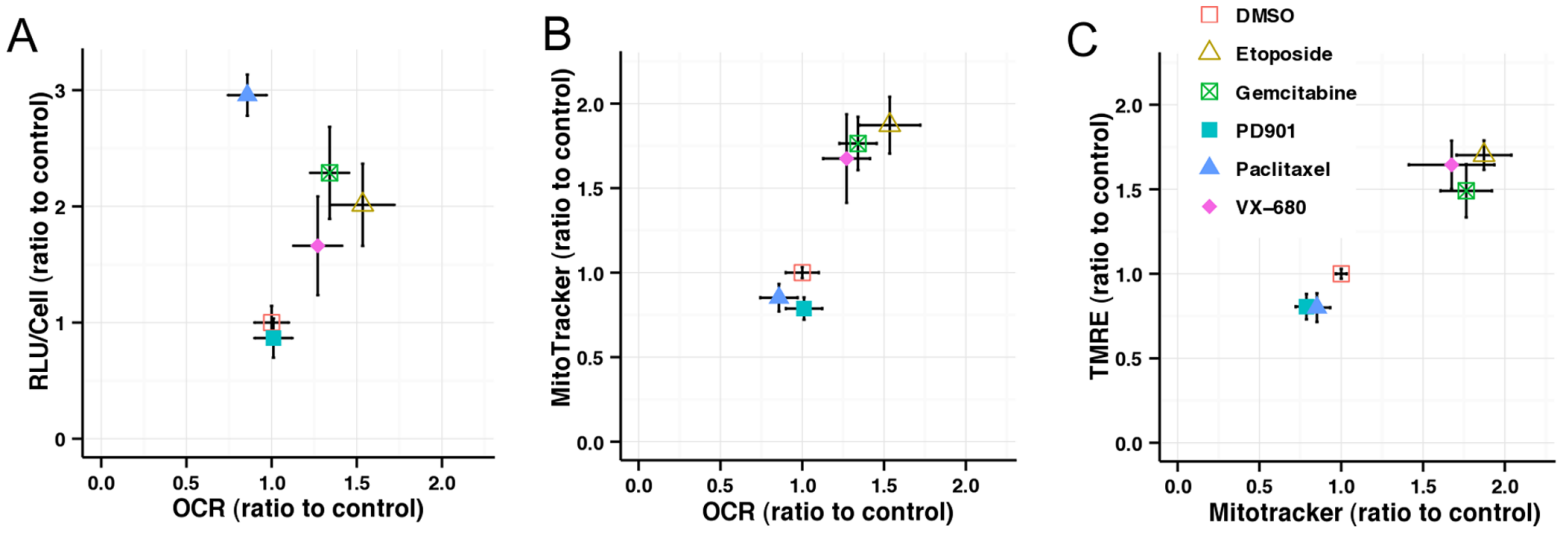
Effects of drug treatment on mitochondrial function. Basal oxygen consumption rate (OCR) determined for cells treated with the indicated compounds (etoposide, 10 µM; gemcitabine 0.1 µM; paclitaxel 0.01 µM; PD901 1 µM, VX-680 0.2 µM) were normalized for cell number. Per-cell OCR is compared with normalized ATP-generated RLU (**A**) and mitochondrial mass (**B**). Cells analyzed for mitochondrial mass by MitoTracker Deep Red staining were also stained with the mitochondrial membrane potential-sensitive dye TMRE, and the mean integrated intensities compared (**C**). All data were normalized as a ratio of the mean DMSO-treated values and are the mean of four replicate wells, error bars show standard deviation.

The baseline OCR data, normalized on a per-cell basis, is plotted against the per-cell ATP and mitochondrial content in figures 7A and 7B. For etoposide, gemcitabine and VX-680 the per-cell OCR changes are very similar to the changes in cell size and mitochondrial mass, whereas the mitochondrial membrane potential does not change significantly (as determined by ratio of TMRE to MitoTracker deep red fluorescence intensity) (Figure 7C). This is consistent with the hypothesis that the increase in ATP per cell for these classes of compounds is due to increased cell size and mitochondrial mass, rather than changes in mitochondrial function. There was no change in the ratio of oxidative to glycolytic metabolism either. Paclitaxel, on the other hand, induced a significant increase in ATP/cell, as described above, without any increase in metabolic activity (both OCR and ECAR were decreased slightly on a per-cell basis). PD901 had the unexpected effect of significantly depressing OCR, despite having no observable effect on mitochondrial or ATP content.

### Time Dependence of Inter-assay Format Variation

A treatment time of 48 hours was chosen for the above studies because it corresponds to ∼2–3 doublings for most of the cell lines, thus the starting number of cells is sufficient to give robust cell count data in the presence of cytostatic drugs, without untreated cells reaching confluence. To examine the extent to which the ATP/cell disconnect is time-dependent, replicate plates were analyzed at different times using etoposide and gemcitabine. Because p53 mutational status is potentially a major determinant of the kinetics and nature of response to treatment with DNA-targeting drugs, we examined A375 (p53 wt) as well as HT29 (p53-null) cells. To maintain suitable cell densities at the assay endpoint, plates were seeded at different densities, then treated for 24, 48, and72 hours before being processed for imaging and ATP assays.

**Figure 8:**
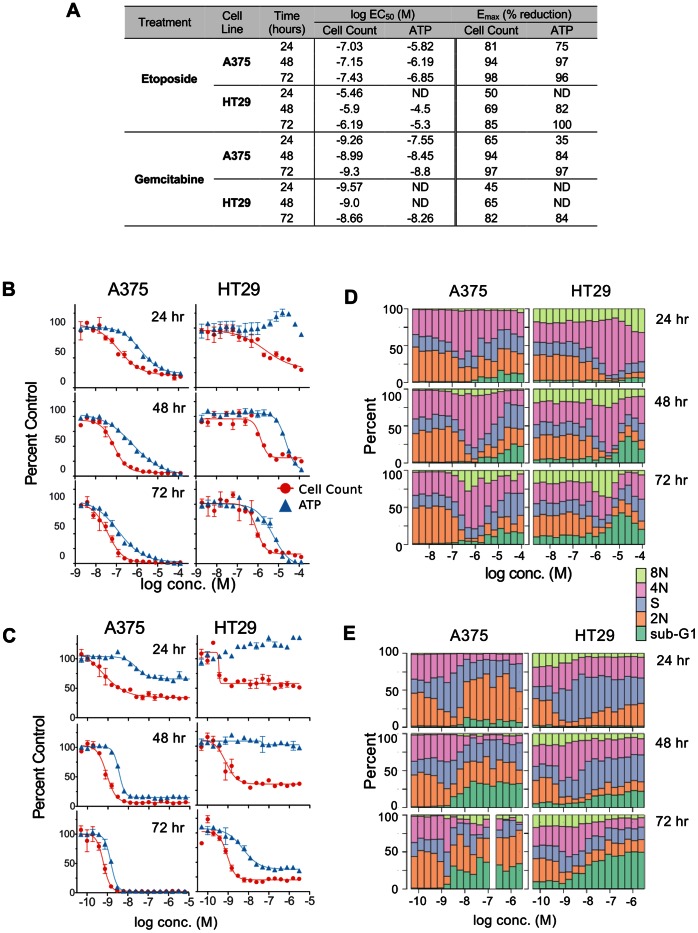
Time-dependence of discrepancies between cell number and ATP assay. A. EC_50_ and E_max_ values HT29 and A375 cells treated with etoposide or gemcitabine for the indicated periods of time were assayed by ATP and high-content assays as described. ND, not determined – no valid curve fit was possible by the standard acceptance criteria described in methods. Assay dose-response curves (**B,C**) and cell cycle profiles (**D,E**) for A375 and HT29 cells treated with etoposide (**B,D**) and gemcitabine (**C,E**).

EC_50_ and E_max_ data for the ATP and cell count dose-responses at the different treatment times are summarized in figure 8A. Figure 8B and 8C show the corresponding dose-response curves for etoposide and gemcitabine. With increasing time there was significantly better convergence of the cell number and ATP curves, with increases in the ATP assay E_max_ values and some left-shifting of the curves. In comparison the cell count EC_50_ values were relatively consistent, while the E_max_ values increased with time. It is noteworthy that HT29 cells treated for 24 hours with gemcitabine or etoposide show an increase above control for the ATP assay signal. While at 48 hours there was not sufficient reduction in HT29 ATP assay signal to give an EC_50_ value, the HT29 ATP response was similar to A375 at 72 hours. The convergence and increased Emax for gemcitabine was associated with an increase in the sub-G1 fraction (figure 8E), suggesting a time-dependent progression from cell cycle arrest to apoptosis. For A375, there was a greater sub-G1 fraction at 48 hours than HT29 cells, which corresponded to a smaller difference between ATP and cell number.

## Discussion

We sought to develop a high-throughput assay to study both antiproliferative potency and mechanism of action of cell cycle-targeting drugs. High-throughput microscopy enables direct counting of cells. Optimization of sample preparation conditions (fixation, permeabilization and staining) and image analysis has enabled a one-step no-wash assay that is also quantitative for DNA content, and hence cell cycle distribution. MitoTracker staining only required an extra reagent addition step, since confocal imaging practically eliminated background fluorescence. The elimination of any requirements for aspiration or washing steps will also greatly facilitate implementation of this assay in 1536-well format.

More complex detection and analysis methods (e.g. immunostaining for cell-cycle regulated proteins and phospho-epitopes, multiple nuclear morphology, texture and intensity parameters) have been used to identify cell cycle sub-populations by high-content analysis (for example [28]–[34]). However we chose to use monoparametric DNA-content binning for several reasons. One of the key objectives of optimizing a no-wash protocol was to ensure that all cells, including detached and fragmented apoptotic cells, are retained, hence immunostaining is not possible. Differentiating G2 from M cells based on nuclear morphology is possible with the cell-level analysis parameter we measured (see for example [22]), but in many cases compound treatment results in abnormal morphologies which do not closely correspond to any of the populations found in untreated cells.

Comparison of direct cell counting with two commonly-used ‘proliferation’ assays that are based on cellular metabolism; ATP content and MTS reductase activity, revealed a common and significant artifact in that, under conditions of cell cycle arrest, the assumed linear relationship between assay signal and cell number breaks down. The average amount of ATP (as inferred from increased Luciferase/Luciferin bioluminescence) or MTS-reducing activity per cell is significantly increased. This increase correlates with, and can be explained by, drug-induced increases in per-cell cell size and consequently mitochondrial content.

In comparison, estimation of viable cell number using total DNA fluorescence (CyQuant assay) was less prone to deviation from the actual cell number. Previous studies have also reported differences in cell number determination between DNA quantification and metabolism-based assays [1], [35], [36]. However, treatments that significantly changed the average DNA content per cell by inducing mitotic arrest and/or endoreduplication also led to an underestimation in the percent change in cell number with the CyQuant assay.

In general, changes in cellular ATP content and MTS activity resulted in one of two types of deviations between cell number and ATP/MTS assay. First, there were cases where E_max_ was greatly reduced, i.e. very shallow dose-response curves. For example gemcitabine reduces ATP signal for HT29 cells by approximately 20%, when in fact cell number has been reduced by 80% relative to control. The other situation is exemplified by etoposide, where the EC_50_ is right-shifted but the dose-response curves converge to similar E_max_ at a sufficiently high concentration. This could lead to underestimation of antiproliferative potency by 10-fold or more. VX-680 presents an intermediate case, where there is a biphasic ATP curve with an intermediate plateau corresponding to an elevated ATP/cell ratio, followed by a second decline. Curves like this fit poorly, if at all, to the standard 4-parameter model and can result in very inconsistent results in terms of both EC_50_ and E_max_.

These different response profiles can be explained by the mechanisms of action of these compounds: There is a biphasic cell cycle dose-response to many of these drugs. First, on-target antiproliferative or cytostatic responses coincide with the reduction in absolute cell number but little cell death. Under these conditions the arrested cells increase in size and mitochondrial content, and correspondingly, the amount of ATP per cell increases. At higher drug concentrations the population phenotype may become less exclusively cytostatic, depending on cell line and treatment time. With increasing late-apoptotic (sub-G1) fraction the average MitoTracker intensity and ATP and MTS per cell declines. For example, for HT-29 cells, aphidicolin and gemcitabine resulted in S or G2 arrest and elevated mitochondrial and ATP content per cell across a wide concentration range, whereas p53-wild type A375 and A549 cell lines underwent a phenotypic switch at higher gemcitabine concentrations, where a greatly increased increased apoptotic fraction correlated with less average ATP per cell. Etoposide on the other hand induced elevation of ATP and MTS activity and mitochondrial mass over a limited concentration range in all cell lines tested. This is consistent with a biphasic mechanisms of action previously observed for these drugs [37], [38]: At lower concentrations repairable DNA damage causes arrest in late S or at the G2 checkpoint with minimal apoptosis and thus accumulation of mitochondria, ATP and MTS activity per cell. At higher concentrations the DNA damage accumulates more rapidly and pervasively, causing arrest and apoptosis earlier in S-phase. A similar pattern of biphasic response explains the two-step curves observed with VX-680, where the predominant phenotype switches from cytostatic endoreduplication to predominantly 4N arrest and cell death, possibly related to off-target activities, at higher concentrations.

The behavior of the MTS assay in the case of the MEK inhibitor PD901 is unusual in that the per-cell level of MTS dehydrogenase activity is increased but the per-cell ATP amount is unchanged by drug treatment. However other kinase inhibitors; VX-680, BI-2536, and crizotinib, also caused a greater discrepancy between MTS assay and cell number than ATP. A similar observation has been reported for imatinib [39] genistein [40], and faslodex [41]. The latter two papers also demonstrated increased mitochondrial activity and mitochondrial mass. Since there are multiple mechanisms and cellular locations of tetrazolium reductase activity [5], the observation that some treatments in this study can result in disconnects between changes in mitochondrial mass and MTS reduction (e.g. PD901) is not unexpected. The mechanism of the biphasic induction by BI-2536 of mitochondrial mass, ATP, and MTS activity at concentrations higher than the fully efficacious antiproliferative concentrations was clearly different from other kinase inhibitors, and warrants further investigation.

There have been a number of reports of chemotherapeutic agents causing increases in mitochondrial mass, including doxorubicin [10] and etoposide [9], [13], [42]. Several different mechanisms have been proposed to explain these increases. Fu et al [9] proposed a direct mechanistic link where activated ATM phosphorylates and activates AMPK, thereby increasing mitochondrial biogenesis. McGowan et al [41] demonstrated that induction of cell cycle arrest by enforced expression of p14ARF resulted in increased mitochondrial mass.

It is worth noting that none of the above reports examined cell size as a factor in changes in mitochondrial content, and therefore were not in a position to differentiate specific increases in mitochondrial biogenesis from baseline mitochondrial proliferation continuing in the absence of cell division. The latter mechanism seems plausible for many of the agents described in the current study. A similar observation was recently published by Kitami et al [43], [44] where a many compounds identified in a screen for increased mitochondrial mass were shown to correspondingly increase cell size.

There has also been a report of microtubule-targeting drugs affecting mitochondrial function via regulation of VDAC activity and ΔΨ by levels of free tubulin [11]. In the current study we also observed an increase in ATP content despite a slight decrease in respiratory activity (both OCR and ECAR) in paclitaxel-treated cells. However we observed increases in cellular ATP levels at E_max_ in response to both microtubule stabilizing and destabilizing drugs, suggesting that the level of free tubulin is not causative. Our data imply that microtubule-targeting agents increase per-cell ATP through a mechanism that is uncoupled from changes in cell size, in contrast to the DNA synthesis-targeting agents and mitotic kinase inhibitors.

While changes in respiratory function and flux clearly control the rate of ATP synthesis, it is less clear when, if at all, changes in flux result in changes in steady-state ATP concentration, which is generally under tight feedback control [45]. The relationship between mitochondrial mass, membrane potential, and cellular ATP levels could also be confounded by variations in contribution of glycolysis to the intracellular ATP pool [46], [47], however with the exception of PD901 we did not observe changes in the OCR/ECAR ratio.

In summary, it appears that there are multiple mechanisms by which different compounds can yield discrepant and misleading results in proxy assays based on energy metabolism, however investigation of specific mechanisms is beyond the scope of the current study.

This study also highlights the fact that the compound mechanisms of action and phenotypic responses frequently do not obey monotonic dose-response behavior. Correspondingly, the discrepancies between absolute cell number and ATP or MTS assay signals can vary greatly depending on the concentration tested. When non-monotonic curves are observed in proxy assays without appreciation of the underlying mechanisms of action, not only is the quality of EC_50_ data compromised but also valuable mechanism-of-action information discarded. There is also potential significant risk of false-negative results when using ATP or MTS assays to either screen compounds for antiproliferative activity, or cell lines for sensitivity to compounds, especially if the compound mechanisms of action and effects on cell cycle, metabolic activity, and survival are not well understood. We show that increasing compound treatment time can reduce the difference between proxy assay readout and actual cell number, by allowing more cells to proceed to apoptosis. However this comes at the cost of losing MoA information. A further rationale for the complementarity of absolute cell count and metabolic proxy assays is demonstrated by the determination of dose-, compound-, and cell line-dependent changes in the ATP/cell value, which can give extra insights in to compound mechanisms of action.

High-content imaging, even simply by DNA staining, thus not only provides more accurate information on the number of viable cells but also provides multiple parameters that offer further insight into compound mechanism of action and heterogeneity of response. The simplicity of the staining procedure and absence of wash steps also makes this approach highly amenable to high-throughput screening and compound profiling in both 384- and 1536-well format.

## Supporting Information

Figure S1 **Dose-response Cell Cycle Profiles.** Cells in the same images used for direct cell counting were classified into five cell cycle bins by integrated DNA intensity. Stacked bars show the relative frequencies of the sub-populations at the indicated concentrations. Each bar is the average of two wells. Black circles indicate relative cell number.
(TIFF)

Figure S2 **Dose-response curves for cell number, ATP and MTS assay signals.** Replicate plates of HT29 cells were treated as indicated for 48 hours then analyzed by ATP or MTS assay or high-content cell counting. **A**. Normalized values for direct cell number (red circles), ATP assay (RLU) (blue triangles) and MTS assays (E_490_) (green squares), lines indicate fits to 4-parameter logistic model. If no line is shown then regression did not result in a curve that met acceptance criteria.
(TIFF)

Figure S3 **Comparison of cell area and mitochondrial content for drug-treated cells.** Variation in cell area and mitochondrial content (integrated MitoTracker deep red intensity) for the same cell populations, normalized as fold change relative to the mean area and intensity of DMSO-treated samples.
(TIFF)

Figure S4 **Metabolic effects of drug treatment.** HT29 cells were treated with the indicated compounds ((etoposide, 10 µM; gemcitabine 0.1 µM; paclitaxel 0.01 µM; PD901 1 µM, VX-680 0.2 µM) for 24 hours before analysis of oxygen consumption rate (OCR) and extracellular acidification rate (ECAR) using the Seahorse XF96 extracellular flux analyzer. Baseline rates (black) were determined at the indicated times before the addition of oligomycin (green) and then FCCP (red). Rate data are normalized to per-well cell number determined by post-analysis high-content imaging.
(TIFF)

Table S1 **Comparison of assay results between formats for a panel of cell lines.** LogEC_50_ and E_max_ data determined by direct cell count, ATP and MTS assays and estimated per-well mitochondrial mass (Mito) for the indicated drugs and cell lines. Color scales indicate deviation from cell count EC_50_, and absolute E_max_. ND, valid curve fits could not be obtained according to the criteria described in Materials and Methods.
(EPS)
