# Supplementary figures and images for: A Simple High-Content Cell Cycle Assay Reveals Frequent Discrepancies between Cell Number and ATP and MTS Proliferation Assays
Source: PLoS One. 2013 May 17;8(5):e63583. doi: 10.1371/journal.pone.0063583 (PMC3656927; doi:10.1371/journal.pone.0063583)

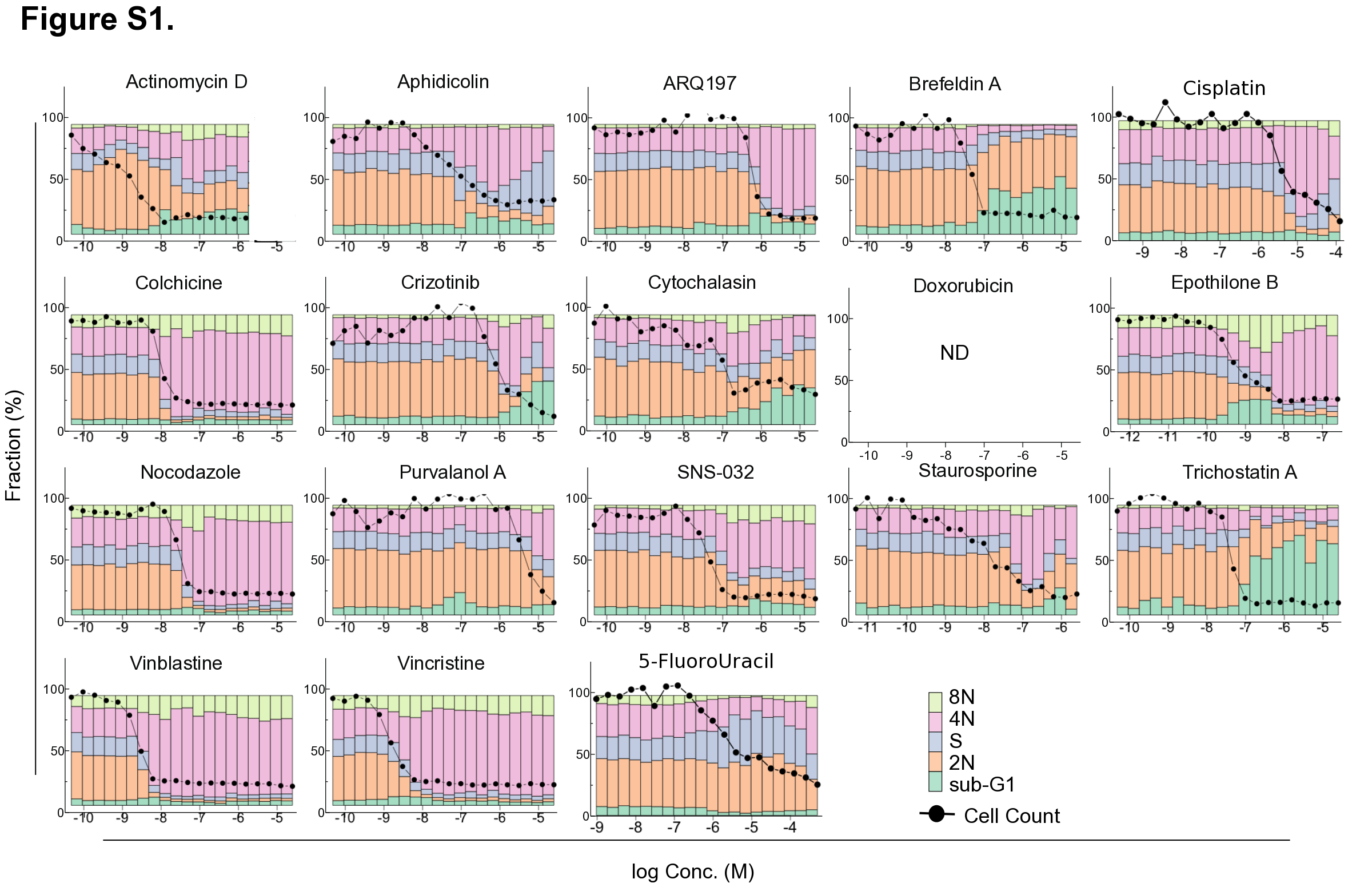

Supplement: Figure S1 — Dose-response Cell Cycle Profiles. Cells in the same images used for direct cell counting were classified into five cell cycle bins by integrated DNA intensity. Stacked bars show the relative frequencies of the sub-populations at the indicated concentrations. Each bar is the average of two wells. Black circles indicate relative cell number. (TIFF) [file pone.0063583.s001.tiff]

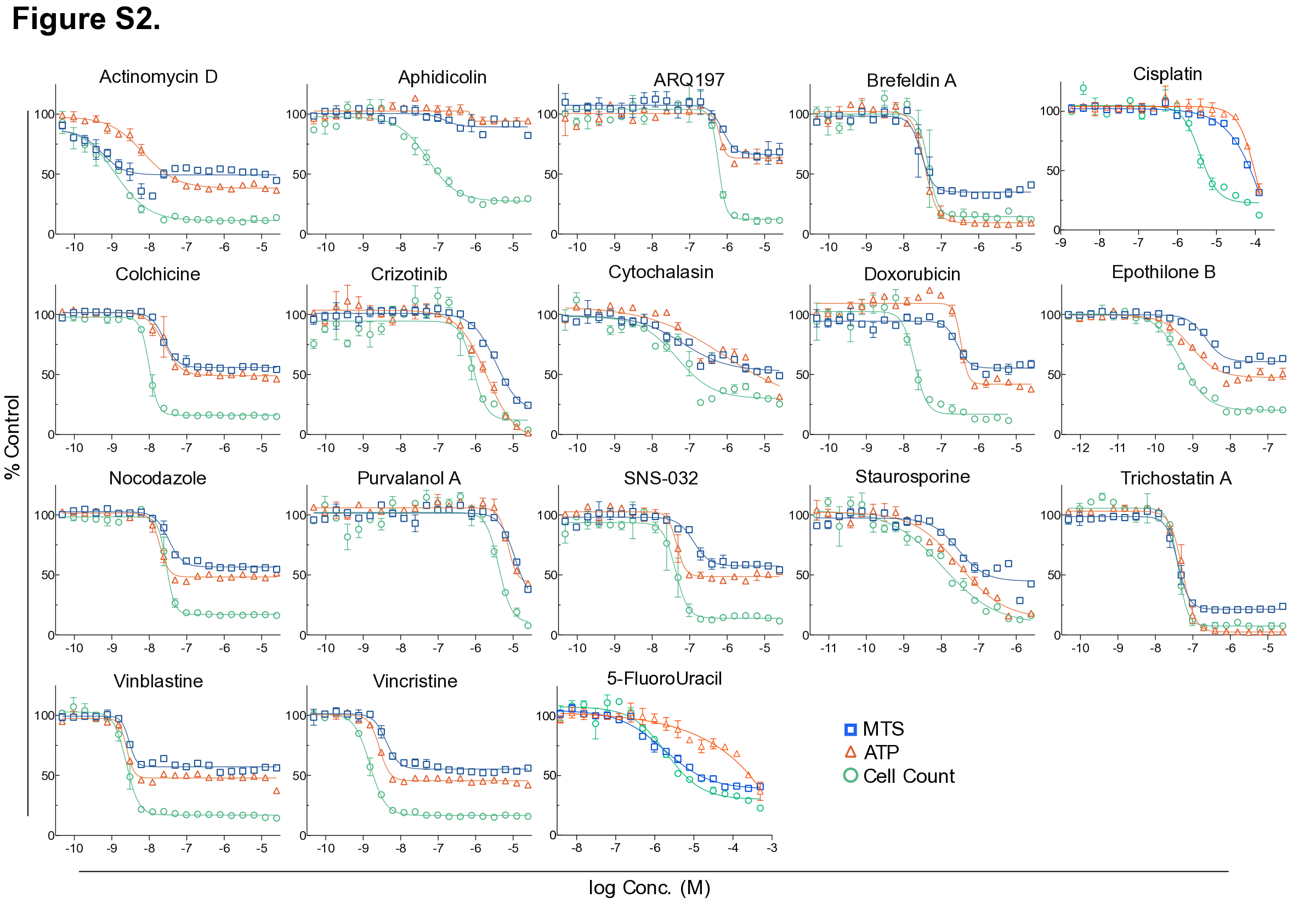

Supplement: Figure S2 — Dose-response curves for cell number, ATP and MTS assay signals. Replicate plates of HT29 cells were treated as indicated for 48 hours then analyzed by ATP or MTS assay or high-content cell counting. A. Normalized values for direct cell number (red circles), ATP assay (RLU) (blue triangles) and MTS assays (E490) (green squares), lines indicate fits to 4-parameter logistic model. If no line is shown then regression did not result in a curve that met acceptance criteria. (TIFF) [file pone.0063583.s002.tiff]

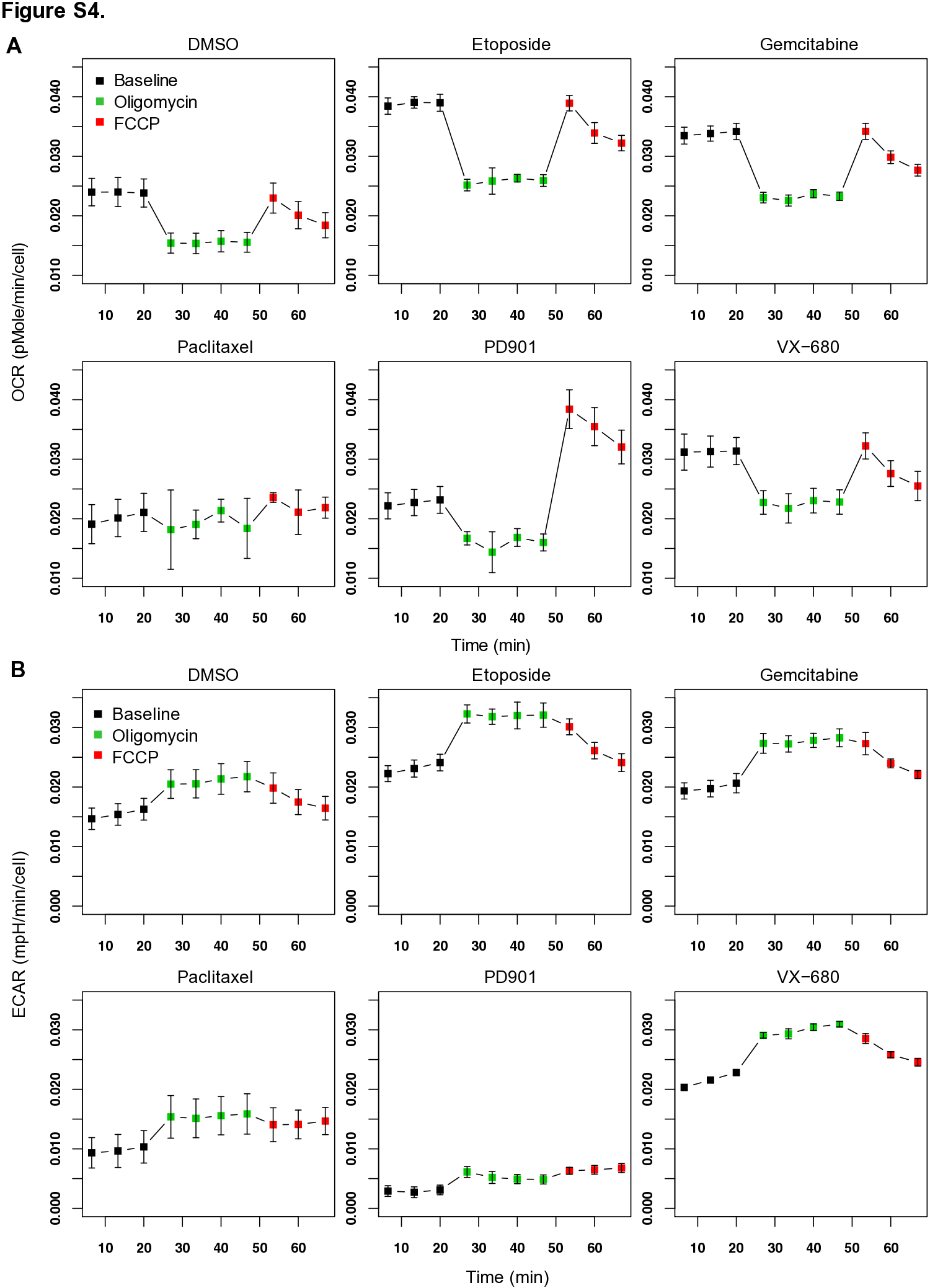

Supplement: Figure S4 — Metabolic effects of drug treatment. HT29 cells were treated with the indicated compounds ((etoposide, 10 µM; gemcitabine 0.1 µM; paclitaxel 0.01 µM; PD901 1 µM, VX-680 0.2 µM) for 24 hours before analysis of oxygen consumption rate (OCR) and extracellular acidification rate (ECAR) using the Seahorse XF96 extracellular flux analyzer. Baseline rates (black) were determined at the indicated times before the addition of oligomycin (green) and then FCCP (red). Rate data are normalized to per-well cell number determined by post-analysis high-content imaging. (TIFF) [file pone.0063583.s004.tiff]
